# Computational drug repurposing based on electronic health records: a scoping review

**DOI:** 10.1038/s41746-022-00617-6

**Published:** 2022-06-14

**Authors:** Nansu Zong, Andrew Wen, Sungrim Moon, Sunyang Fu, Liwei Wang, Yiqing Zhao, Yue Yu, Ming Huang, Yanshan Wang, Gang Zheng, Michelle M. Mielke, James R. Cerhan, Hongfang Liu

**Affiliations:** 1 Department of Artificial Intelligence and Informatics Research, Mayo Clinic, Rochester, MN USA; 2 Department of Preventive Medicine, Northwestern Medicine, Northwestern University, Chicago, IL USA; 3 Department of Health Information Management, School of Health and Rehabilitation Sciences, University of Pittsburgh, Pittsburgh, PA USA; 4 Department of Laboratory Medicine and Pathology, Mayo Clinic, Rochester, MN USA; 5 Wake Forest University School of Medicine, Winston-Salem, NC USA

**Keywords:** Drug development, Computer science, Virtual drug screening, Computational models

## Abstract

Computational drug repurposing methods adapt Artificial intelligence (AI) algorithms for the discovery of new applications of approved or investigational drugs. Among the heterogeneous datasets, electronic health records (EHRs) datasets provide rich longitudinal and pathophysiological data that facilitate the generation and validation of drug repurposing. Here, we present an appraisal of recently published research on computational drug repurposing utilizing the EHR. Thirty-three research articles, retrieved from Embase, Medline, Scopus, and Web of Science between January 2000 and January 2022, were included in the final review. Four themes, (1) publication venue, (2) data types and sources, (3) method for data processing and prediction, and (4) targeted disease, validation, and released tools were presented. The review summarized the contribution of EHR used in drug repurposing as well as revealed that the utilization is hindered by the validation, accessibility, and understanding of EHRs. These findings can support researchers in the utilization of medical data resources and the development of computational methods for drug repurposing.

## Introduction

It takes an average of 13 years and 2–3 billion dollars to bring a new drug from bench to bedside, with the process comprising examination of its efficacy, toxicity, and pharmacokinetic and pharmacodynamic profiles in cell- and animal-based studies to safety and efficacy in human subjects in clinical trials^1^. The escalating cost and length of time make drug development a less desirable business for investment^2^. Drug repurposing, on the other hand, aims to discover new medical indications for an approved or investigational drug. As the dosing and safety of the drug are well studied, clinical trials can be accelerated, significantly reducing the development time and cost. There are some successful examples of drug repurposing, such as the usage of metformin for various cancers^3^, sildenafil citrate for erectile dysfunction, and thalidomide for erythema nodosum leprosum (ENL) and multiple myeloma.^2^

The most critical task of drug repurposing is to identify new associations between drugs and diseases. Traditional biomedical experiments are based on binding assays and phenotypic screening, which are expensive and time-consuming. Conversely, in-silico methods gain their popularity through the analysis of heterogeneous data based on Artificial intelligence (AI) methods^4^, such as genetic association analysis, pathway mapping, molecular docking, and signature profile matching^5^, as such methods allow for all analysis to be done computationally in a time and cost-efficient manner. Computational drug repurposing can utilize a diverse set of data resources, including omics data (e.g., gene and protein expression)^6^, biomedical association/relation knowledgebase^7^, biomedical literature^8^, and the electronic health record (EHRs)^6^. Big EHR datasets offer a real-world perspective rooted in clinical care that provides rich longitudinal diagnostic and pathophysiological patient data, which can facilitate the generation and validation of drug repurposing hypotheses (e.g., statistical significance)^3^. The unique capability of incorporating EHR-based data into repurposing methods is the ability to test a large number of drug repurposing hypotheses in parallel by identifying the cohorts that either have or have not been prescribed a particular medication using large patient populations followed for several years^9^. For this reason, EHR-based drug repurposing has been identified as a unique, cost-effective opportunity by the drug development field, and a diverse set of applications have been proposed, including phenome-wide association studies (PheWAS) based on statistic tests^10–12^, similarities based on disease network^6,13^, and association rule-based interaction mining^14^.

In this survey, we reviewed current computational drug repurposing approaches utilizing EHR-based data. We retrieved 1145 combined results containing 1370 publications from four databases (i.e., Embase, Medline, Scopus, and Web of Science) between January 2000 and January 2022, in which 33 articles were included in the final review. Compared to the existing surveys^5,13,15,16^, we systematically investigated the relevant studies from four perspectives, (1) publication venue, (2) data types and sources, (3) method used for data processing and drug repurposing prediction, and (4) disease targeted, the validation for the experiment, and tools released. We learned that, compared to validation, EHR is mainly used for building the predictive models, where drug effects on laboratory tests, drugs used for diseases, genetic mutations related to diseases, and disease-laboratory tests associations were the most popular data used among all the studies. While EHR datasets have gained popularity in drug repurposing, the utilization is hindered by the validation (e.g., discoveries are unverifiable using other available knowledge derived from published literature and clinical trial and application), the accessibility (e.g., the release and sharing of datasets and tools due to patients’ privacy), and understanding (e.g., adaptations of NLP tools and standardization). This study enabled us to gain a more concrete understanding of how the EHR is utilized in a drug repurposing context and to provide potential guidelines for designing the EHR-based drug repurposing methods.

## Results

We abstracted 33 articles based on four themes with the following flow, (1) journal and articles, (2) data used, (3) methods, and (4) results of repurposing. During the process for each theme, important data elements were identified by the first author and validated by all seven reviewers. The synthesis of the articles for each data element was conducted by each reviewer. The results were finally validated and organized by the first author. The disagreements in synthesis were resolved among all the reviewers in the consensus meeting. The general summarization of the articles is shown in Supplementary Table 1. The flow details for each article can be found in Supplementary Method 1.

### Publication venue

**Fig. 1:**
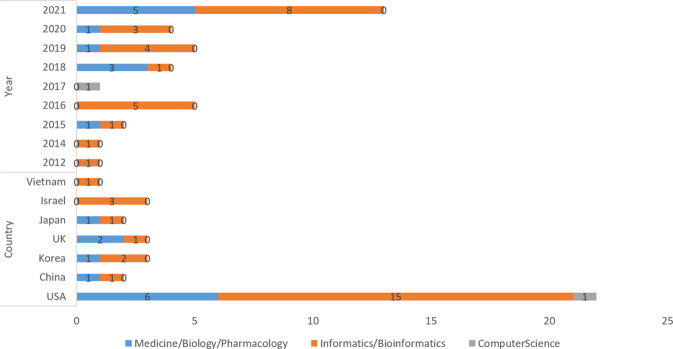
Distribution of publication type, stratified on the year of publication and country of origin.

The 33 papers reviewed consisted of 29 journals and four conference articles (see Fig. 1). We manually categorized those articles into three types: (1) Computer Science, (2) Informatics/Biomedical Informatics, and (3) Medicine/Biology/Pharmacology. The majority of the articles were Informatics/Biomedical (*n* = 22) and Medicine/Biology/Pharmacology (*n* = 10). We also noticed that the conference articles were Informatics/Biomedical, suggesting this topic or methodology is more popular among the Biomedical Informatics community. Most studies were conducted in the United States (*n* = 22), with the remainder being scattered amongst Asian and European countries. In addition, the topic of EHR-based drug repurposing gains popularity from the year 2012 (*n* = 1) to 2021 (*n* = 10).

### Data

The majority of studies relied on the EHR from an institution affiliated with either the authors themselves (e.g., Vanderbilt University Medical Center^17–19^) or one of their collaborators (e.g., Mayo Clinic^18,19^). Only three studies utilized publicly available datasets, including IBM Watson Health Explorys database^20^, MIMIC-II^21^, and adverse event reporting systems (AERSs)^22^. Most of the studies used only EHR, while others utilized multiple kinds of sources to facilitate the drug repurposing, such as knowledge bases (*N* = 11), Omics databases (*N* = 7) (Please note, we distinguished Omics data from EHR data) (see Supplementary Fig. 2a). Among all the association knowledge bases, drug-gene information was the most popular (see Supplementary Fig. 2b). Drugbank^23^ was the main source of drug-gene (protein) information in the studies (*N* = 11). We noticed widespread usage of biomedical repositories, such as Sider^24^ for side effect information (*N* = 3) and OMIM^25^ for gene-disease relations (*N* = 2).

**Fig. 2:**
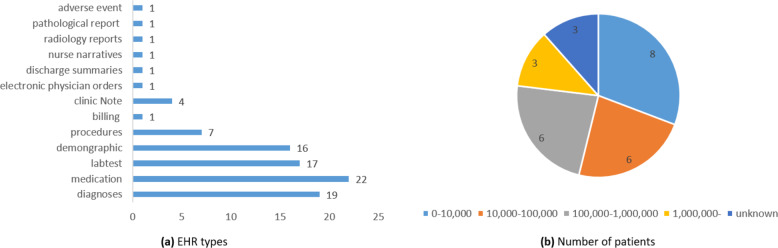
Distribution of EHR types and number of patients. **a** shows the distribution of the EHR types, and **b** shows the distribution of the number of patients.

Amongst all the 22 EHR data types covered in our survey, medication (*N* = 22), diagnosis (*N* = 19), lab test (*N* = 17), and demographic (*N* = 16) were the most frequently used (see Fig. 2a). Most studies were conducted based on patient cohort sizes of less than 10,000 (*N* = 8), 10,000 to 100,000 (*N* = 6), and 100,000 to 1,000,000 (*N* = 6) (see Fig. 2b). We note that a few studies did not specify the size of the patient cohort used (*N* = 3). Supplementary Table 2 shows the detailed information of the data in the reviewed studies.

### Drug repurposing methods

**Fig. 3:**
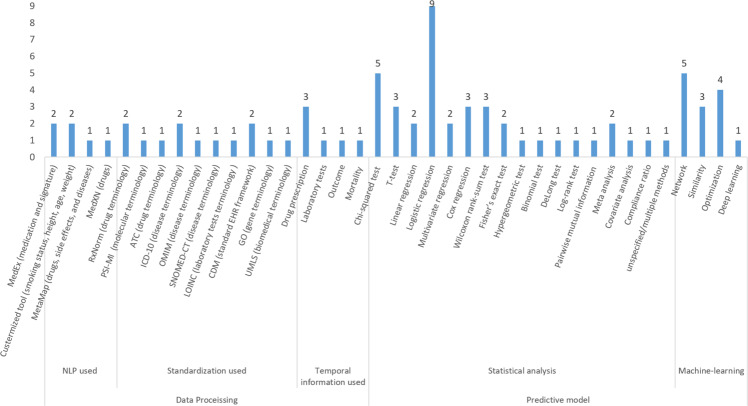
Distribution of different data processing methods and the predictive model.

Figure 3 shows the number of papers with different data processing methods for EHR data regarding natural language processing (NLP), standardization, or temporal data processing. Of the surveyed studies, five studies utilized NLP to process their data, seven used standardization, and four dealt with temporal data. We note that three studies implemented more than one data processing method (e.g., MedEx and RxNorm CUI were used to extract and standardize medication information^19^, and the Observational Medical Outcomes Partnership Common Data Model (OMOP CDM) was used for both drug prescription and laboratory tests^26^).

NLP pipelines were used to extract a diverse set of information that differed depending on individual study needs. For example, drugs and diseases were extracted from triads of sentences by using MetaMap^21^. Regarding the adoption of standardization methods, standardization efforts are mainly focused on using standard terminologies for medical concepts. For example, Proteomics Standard Initiative—Molecular Interactions (PSI-MI) codes were used for proteomics, Gene Ontology (GO) for genomics, Anatomical Therapeutic Chemical (ATC) codes for drugs, and ICD-10 and Online Mendelian Inheritance in Man (OMIM) for disease data^27^. Similarly, Systematized Nomenclature of Medicine-Clinical Terms (SNOMED CT) was also used for diseases, RxNorm for drugs, logical observation identifier names and codes (LOINC) for Laboratory tests^20^, standard billing codes for clinical phenotypes^28^, and Unified Medical Language System (UMLS) for multiple kinds of biomedical related concepts^29^. Temporal information was primarily used to track disease progression. For example, temporal data was used in one study to analyze the association between the virological status of patients and all-cause mortality as well as other individual-level factors^30^. Supplementary Table 3 shows the summary information of data processing in the studies.

As shown in Fig. 3, statistical analysis and machine learning are two predominant computational approaches used for drug repurposing through mining a large set of health data. In statistical analysis methods, statistical models and tests are used to determine the effect of drugs on disease targets or other related clinical variables such as genes and laboratory tests. For example, Wang et al.^31^ searched drug and gene information from public pharmacological and genomic databases as well as private EHRs for glaucoma diseases. It used *p* values based on the chi-square tests and false discovery rates (FDR) of drugs targeted to glaucoma genes/diseases to detect potential treatment candidates. For example, the prevalence of glaucoma was 0.11% in theophylline-treated patients, and 0.058% in celecoxib-treated patients, suggesting these drugs may have antiglaucoma effects as the incidence of glaucoma was significantly lower in these drug-use cohorts than in healthy individuals. Wu et al.^19^ classified a patient cohort into two comparison groups—an exposure group with drug prescription and a non-exposure group without drug prescription and applied cox regression to measure the association of drugs with cancer survival for suggesting repurposing candidates. Goldstein et al.^17^ used logistic regression (or multivariate regression) and derived *p* values to examine the association between drug candidates and genetic mutation (or glucose tolerance test) data for identifying drug repurposing candidates for gestational diabetes.

Machine learning is another type of common computational approach for predicting new disease targets of existing drugs. Three popular machine learning methods are based on similarity/interaction network, the least-square optimization method, and deep learning. For instance, Zhou et al.^20^ developed a network-based prediction system of disease-target interactions by modeling phenotypic and genetic relationships among drugs, side effects, diseases, and genes for identifying repositioned drug candidates. Ghalwash et al.^32^ formulated the problem of finding drugs that have an effect on the levels of laboratory test results as a regularized least-square unconstrained convex optimization problem. Liu et al.^33^ created a high-throughput screening framework with existing large-scale real-world data. The framework extracted potential repurposing drug ingredients, identifies the corresponding user and non-user sub-cohorts, computes features and disease progression outcomes for all patients in both sub-cohorts, and estimates the treatment effects using deep learning methods. Supplementary Table 4 summarizes the computational methods in detail.

**Fig. 4:**
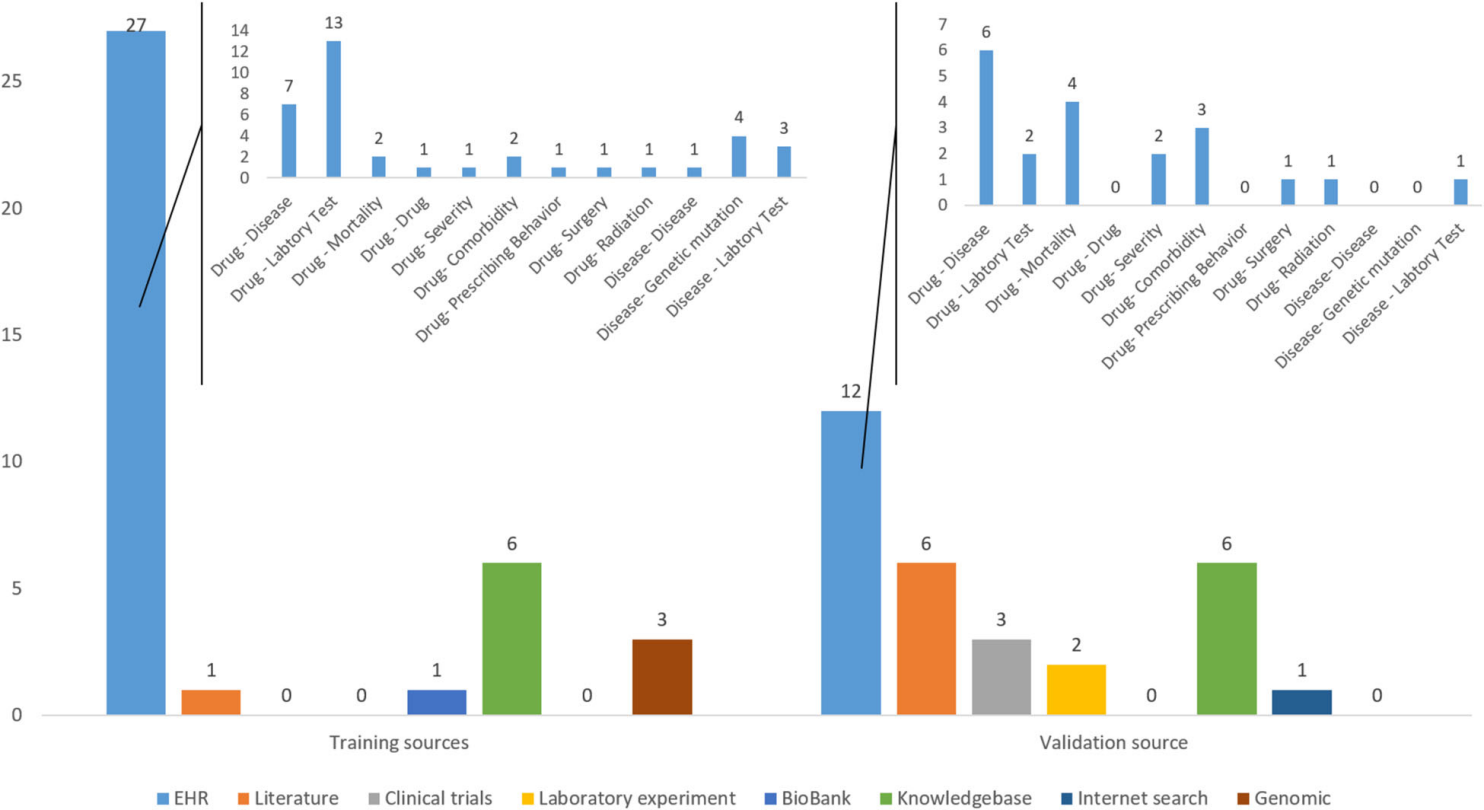
Distribution of resources for training and validation.

Evaluation of EHR-based computational drug repositioning research is critical to ensure valid and reliable computation methods and new signals. Unlike predictive modeling or adverse drug reaction detection, where the gold standard outcomes can be well defined, there may be a lack of well-established evidence or ground truth to validate the newly discovered target signals in drug repositioning research. Therefore, the evaluation may rely on multiple internal and external sources of evidence. Figure 4 summarizes the sources for training and validation. Of the 33 papers reviewed, six did not present any methods for assessing drug performance. Of the 27 that did, the most common performance metrics used were machine learning related (e.g., precision-recall, AUC-ROC). Risk ratios (e.g., hazard, odds, and relative risk) were commonly reported to evaluate the effectiveness of candidate drugs. With respect to validation, 12 papers performed validation of any hypothesis candidate drugs against other data sources based on EHR data, ten based on biomedical literature, and nine based on public knowledge bases.

The EHR is the most frequently reported source for training and validation since it contains rich, dense, and longitudinal information. The drug effects on laboratory tests were mainly used in building predictive models (*N* = 12). For validation, Drug-Disease information (*N* = 6) observed in the EHR is mainly used. Please note, that a dataset can be both used for training and validation. The validation can be conducted by retrospectively analyzing EHR data to estimate the usage and effects of the candidate drug. For example, Wang et al. searched EHR data to obtain information on the usage of the candidate drugs and glaucoma^31^. Due to potential issues of data quality or information representation (e.g., unstructured text), manual chart reviews are often required when leveraging EHR for evaluation. Cai et al. conducted a chart review of EHRs based on randomly selected 20 participants to determine the accuracy of newly identified phenotypes^34^. In addition to EHRs, external databases such as Drugbank and clinical trials databases can be great resources for evaluation purposes. One common way of leveraging these databases is through study replication, a method by which target associations are reproduced using the same computational methods on a different dataset, and the difference in the study outcomes is statistically compared. Cai et al.^34^ used two additional external data sources BioVU and UK Biobank to cross-examine the association between a genetic variant and coronary heart disease phenotypes. Xu et al.^35^ performed a comprehensive performance comparison to the existing state-of-the-art drug repositioning methods to reveal the advantages of the proposed methods. Out of 33 articles, two studies^27,35^ conducted an additional laboratory study to validate the potential therapeutic effect on animal models and demonstrated additional validity to the proposed methods.

Due to the lack of ground truth and potential EHR-related data quality issues, we recommend having multiple evaluations on different data sources. We found that 13 out of 33 studies reported more than one evaluation method. For example, Wu et al.^19^ incorporated two different validation methods: (1) supporting evidence from biomedical literature, and (2) supporting evidence from human interventional cancer trials. Paik et al. ^27^ used computational evaluation (tenfold cross-validation) on known associations in a vivo zebrafish model of ALS. Hsieh et al. ^36^ validated the candidate drugs through both in vitro drug screening and real-world population-based studies leveraging EHRs. Supplementary Table 5 summarizes the validation methods in detail.

### Drug repurposing results

**Fig. 5:**
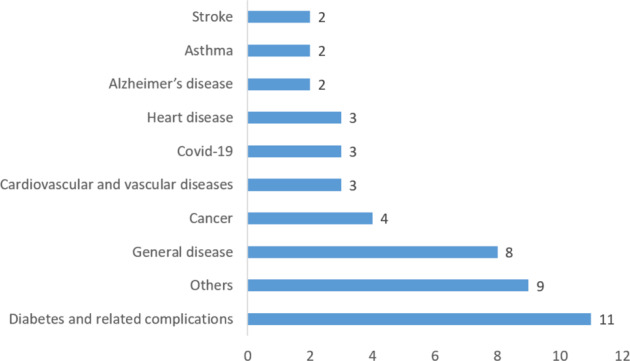
Distribution of diseases targeted.

Figure 5 shows the disease targeted. The most common repurposing target was diabetes-related, consisting of 10 out of the 33 publications^17,26,29,32,37–42^, including type 2 diabetes^37,41,42^, gestational diabetes^17^, diabetes (unspecified)^26,29^, diabetes-related tests including glycated hemoglobin^32^ and Fasting Blood Glucose^38–40^. Six publications did not focus on any specific diseases^21,22,27,28,43,44^. For example, Dang et al.^21^ aimed to establish a generic process and method to integrate phenomic data in EHR with omic and drug data.

Cardiovascular-related diseases are also focused on in seven publications. Specifically, Jang et al.^41^ targeted congestive heart failure, myocardial ischemia, and stroke, Ghalwash et al.^32^ targeted low-density lipoprotein, which is a risk factor for cardiovascular and vascular diseases, Kim et al.^26^ targeted dyslipidemia, Cai et al.^34^ targeted cardiovascular disease, Liu et al.^33^ targeted coronary artery disease, Nordon et al.^29^ targeted hypertension, and 366 targeted coronary heart disease, congestive heart failure, heart attack, and stroke. In addition, there are four publications targeting at cancer^18,19,35,45^, three targeting at COVID^36,46,47^, two targeting at asthma^41,42^.

It is worthwhile to note that some of the reviewed articles did not report on specific drugs, but rather presented a selection of top *n* repurposed drug candidates as determined by their respective methodologies. Of those that did subset reported drugs, they were typically sub-selected by certain drug types, such as statins, triptans, PPIs, and nasal steroids in one study, α1‐adrenoceptor antagonists in another, and antihypertensive calcium channel blockers in a third. Of the 33 studies reviewed, only five reported results focused on a single drug, metformin in the case of Xu et al.^18^, febuxostat in the case of Muraki et al.^48^, terbutaline sulfate in the case of Paik et al.^27^, Fluoxetine in the case of Bi et al.^45^, and Dextromethorphan in the case of Cummings et al.^49^. Intuitively, this finding makes sense as most methods are focused on presenting candidates for further screening rather than having a pre-existing drug that should be further studied, and as such methods would result in a selection of candidates that should then be cross-validated against known clinical indications for method validity, rather than a clinical validation of an individual drug itself selected from said list of candidates. Supplementary Tables 1, 6 show the drugs explored and the corresponding diseases targeted in detail.

### Data and tools published

Despite the widespread and vital use of EHR data for drug repurposing research, datasets and tools were not readily electronically available to the public in many of the surveyed studies. Table 1 shows the publically shared data and tools among the reviewed studies. Only 1 study^33^ of the 33 reviewed studies can be fully reproducible with publically available EHR and the tool so as to verify the original studies to follow-up studies. In terms of the dataset, seven of these studies used publicly open EHR datasets: IBM Watson Health Explorys database^20,50,51^, IBM Health MarketScan database^33,45,51^, and MIMIC-II^21^, which are the only research that shared their original dataset, Vanderbilt Synthetic Derivatives database^17^, through data use agreement. From the perspective of sharing developed tools, five studies shared their own tools^28,29,33,36,42^. In contrast, others indicated open software, which they used, without their practical implementations^20,27,45^. Lastly, some of the studies shared the analysis and results in the form of supplements or separated links^19,26,27,31,33,35,36,41,42,44–47,49–52^.

**Table 1:** Summary of publically shared data and tools.

Paper	Type	Description of resource	Link
Goldstein et al. ^17^	EHR	BioVU of Vanderbilt University Medical Center	https://www.vumc.org/dbmi/biovu
Zhou et al. ^20^	EHR	IBM Watson Health Explorys database	https://www.ibm.com/products/explorys-ehr-data-analysis-tools
Dang et al. ^21^	EHR	Medical Information Mart for Intensive Care - II	https://mimic.physionet.org/
Zhou et al. ^50^	EHR	IBM Watson Health Explorys database	https://www.ibm.com/products/explorys-ehr-data-analysis-tools
Bi et al. ^45^	EHR	IBM Health MarketScan database	https://www.ibm.com/products/marketscan-research-databases
Liu et al. ^33^	EHR	IBM Health MarketScan database	https://www.ibm.com/products/marketscan-research-databases
Ozery-Flato et al. ^51^	EHR	IBM Watson Health Explorys database IBM Health MarketScan database	https://www.ibm.com/products/explorys-ehr-data-analysis-tools https://www.ibm.com/products/marketscan-research-databases
Challa et al. ^28^	Tool		https://github.com/judytlewis/drugRepurposing
Hsieh et al. ^36^	Tool		https://github.com/yejin jkim/drug-repur posing-graph
Liu et al. ^33^	Tool		https://github.com/ruoqi-liu/DeepIPW
Nordon et al. ^29^	Tool		https://github.com/TechnionTDK/repurposing
Wen et al. ^42^	Tool		https://github.com/HoytWen/CCMDR

## Discussion

EHRs are an invaluable source of large-scale clinical data capable of simulating drug repurposing strategies in an uncontrolled, real-world environment as opposed to the controlled environment of clinical trials, which is one of the biggest benefits of drug repurposing. EHRs were gaining popularity in diverse applications for drug repurposing, such as COVID 19^53^, or the upstream applications (e.g., providing a support/input resource for other applications, medical cost reduction^43^) and downstream applications (e.g., utilizing the results from other applications as an input). In this paper, we systematically reviewed the literature published between 2000 and 2022 to better understand how EHR datasets are directly utilized to facilitate drug repurposing. In the course of our review, we noted the following considerations when applying EHR data to drug repurposing tasks:

Firstly, as the real-world evidence (RWE), EHRs provide clinical evidence with a heterogeneous set of healthcare data captured outside the existing paradigms and standards of the drug development process, which has tremendous value by improving the applicability of the results to a real-world environment^54^. As such, more than 90% of pharmaceutical companies make RWE investments across the entire life cycle in drug development^55^. Compared to pharmaceutical companies, research organizations have limited resources to leverage heterogeneous healthcare datasets from multiple sources. The risk of biased results caused by the limited EHR drives the academic organizations to focus more on the studies in a controlled environment (e.g., clinical trials), which is the main reason that we have only 33 articles fitting the inclusion criteria.

Secondly, a substantial (50%) proportion of reviewed papers performed their validation tasks via cross-referencing data, either against the EHR itself when the hypothesis generation methodology did not incorporate that data, or other data sources such as biomedical literature, clinical trials, public knowledge bases, and drug information datasets when it did. Fundamentally, this suggests that the knowledge contained in any one of these data sources is likely to be present within some amalgamation of the others. This observation reflects the reality of the EHR itself—the EHR contains a record of clinical decision making, which, with some rare aberrations, is typically reflective of contemporaneous best practices which are themselves reflections of known medical knowledge derived from published literature and empirical observations and evaluated through clinical trial and application. It is therefore rare that a hypothesis generated by some in-silico EHR-based framework will be unverifiable using other available knowledge—instead, we postulate that the value in bringing in EHR data for drug repurposing tasks lies in its reflection of real-world considerations such as evaluating and alleviating the impact of socioeconomic determinants of health on drug choices and identifying alternative therapies, as opposed to other knowledge sources which are typically conducted in more controlled environments (particularly those studies involving pure physical/chemical simulation).

Thirdly, the biggest issue of EHR-based methods is the inaccessibility of any data involved^56^. Thus only primary academic medical centers can fully leverage any advantages of utilizing EHR-based methods. While sharing data and tools across collaborating healthcare providers or institutions as a small group may be feasible while the study is ongoing, there is a rare movement to deposit the data and tools for public usage. The main barrier is rooted in protecting the privacy of patient health data. Therefore, significant burdens and efforts are additionally required for researchers to provide freely available resources. For example, additional funds and efforts are required to perform the de-identification process of the given EHR (e.g., MIMIC-II^21^) or further ongoing management to process restricted data use agreement (e.g., Vanderbilt Synthetic Derivatives database^17^) is essential. Furthermore, advanced data processing methods (e.g., NLP) are needed for handling unstructured data, different data formats, bias (e.g., more expensive drugs are more likely to be prescribed like lenalidomide for multiple myeloma)^3^, and missing data^5^. Standardization of EHR (e.g., Fast Healthcare Interoperability Resources and OMOP Common Data Model) to represent data elements in a standardized format (e.g., terminologies or coding systems) may be a good investment to support computational pipelines.

Lastly, EHR provides large longitudinal medical records in clinical settings, which improves the applicability/reliability of the predictive models. However, temporal information is seldom incorporated into the experiment. The data model that incorporates temporal information will reduce the bias in the validation. Another issue of EHR-based methods is that they cannot reveal causality^56^. More mechanisms (e.g., biological pathways related to drug targets) need to be studied to better understand the toxicity and tolerability of drugs in humans. The leverage of genetic information provides another potential to improve the performance and interpretability of drug repurposing. For example, both the eMERGE network and the All of US Initiative link EHR to genetic information for multisite studies^11^. The integration of EHR and patients’ genetic information will increase the number of features (e.g., phenotypes and genotypes) to further promote the development of drug repurposing.

There are a few limitations in this review that must be mentioned. Firstly, while the authors tried their best to conduct a comprehensive review, the authors acknowledge that some bias may still exist in the selection, filtering and review of the papers due to the perspectives and backgrounds of the authors. Additionally, some related articles published may not be included due to the selection of search strings, databases, and language. For instance, in some studies^57–59^, where usage of EHR is not explicitly mentioned in the text and inferred by a human reader, thus causing them to not be retrieved by our search query. Secondly, this review only focused on the studies that directly used EHR for drug repurposing, and studies that used EHR (e.g., a follow-up study based on a study utilizing EHR to explore epilepsy and twelve autoimmune diseases^60^) indirectly to facilitate drug repurposing are excluded. We acknowledged that some studies excluded may be potentially important and provide new methods of leveraging EHR. It is worth noting that the scope of our review is limited only to the methodologies involved in drug repurposing utilizing EHR data. There are, however, other factors that can affect the viability and efficiency of such methods, including questions surrounding the source EHR data itself, such as how patients relevant to the studies in question are identified, computational representation of EHR data, and varying methods to render EHR data more computationally accessible, such as natural language processing. While studies on these topics have been excluded from our review due to being out of scope, their importance to the overall topic cannot be understated, and we would encourage readers to further review existing works on the different applications of EHR (e.g., refs. ^61–64^) as such topics will have a profound impact on the feasibility and performance of many of the methodologies discussed in this review. In addition, while there are sub-topics in EHR-related studies (e.g., EHR data harmonization, high dimensionality, confounding adjustment, patient matching) critical to our survey, we are unable to discuss them in our manuscript as those topics are not sufficiently presented in the reviewed papers. We further suggest the readers refer to the surveys on those topics (refs. ^65–68^) for a more comprehensive discussion.

## Methods

We followed Preferred Reporting Items for Systematic reviews and Meta-Analyses extension for Scoping Reviews (PRISMA-ScR) guidelines^69^ to perform our review. We conducted a thorough search which was restricted to the full-length research articles published in journals and conference proceedings from four databases (Embase, Medline, Scopus, and Web of Science) published within 22 years (i.e., January 1, 2000, to Jan 10, 2022). We only included the original research articles written in English and excluded those in the forms of review, abstract, poster, podium, commentary, perspective, note, and editorial. The detailed keywords used as tailored for each database are provided in Supplementary Note 1.

**Fig. 6:**
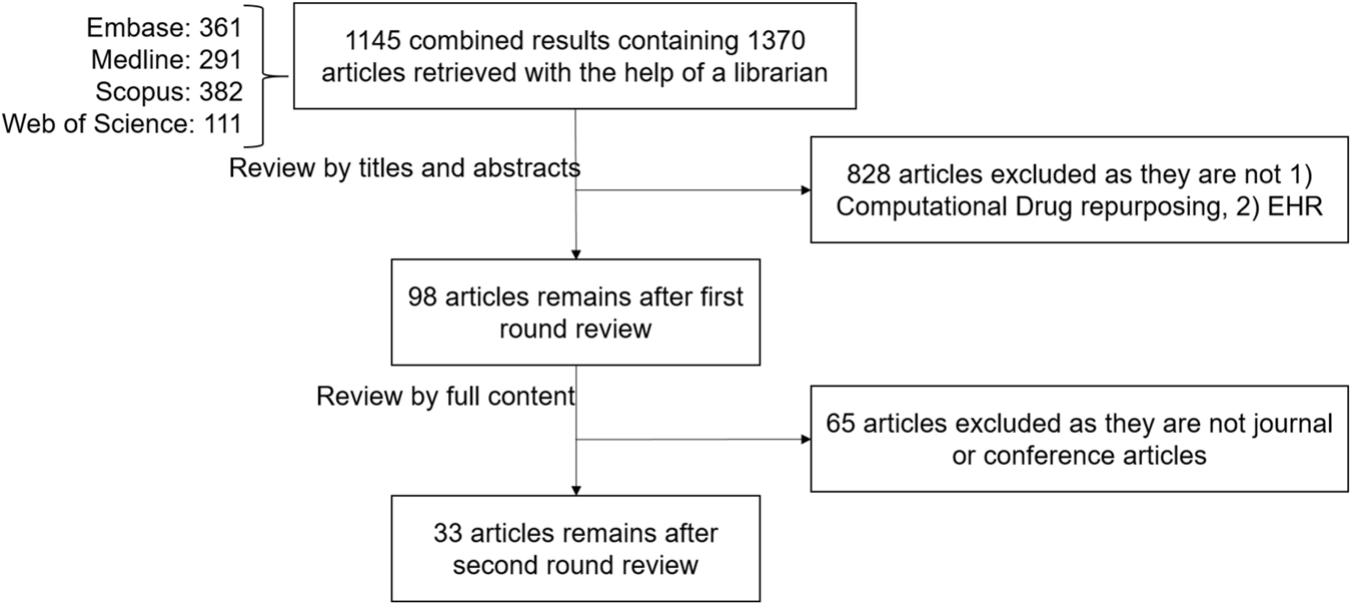
Flow chart for article selection and filtering.

Our literature search obtained 1145 combined results that consisted of 1370 distinct articles (one result may contain multiple articles in one conference proceeding). Seven reviewers (Y.Z., S.M., S.F., L.W., M.H., Y.Y., and A.W.) independently reviewed the titles and abstracts of these articles and filtered out those studies which are (1) not computational drug repurposing, (2) did not utilize EHR data in the modeling or experimenting, (3) are not full research article (e.g., conference and journal) with two round reviews. The excluded articles were double-checked by the first author, NZ. Any disputation of the exclusion is discussed and resolved among the reviewers. This survey focuses on the remaining 33 research articles. A flow chart of how the articles were filtered and reviewed is shown in Fig. 6.

## Supplementary information

Supplementary files

PRISMA Checklist

## Data Availability

Any data generated or analyzed are included in this article and the Supplementary Information files. Aggregate data analyzed in this study are available from the corresponding author on reasonable request.
