# Supplementary material for: Computational drug repurposing based on electronic health records: a scoping review
Source: NPJ Digit Med. 2022 Jun 14;5:77. doi: 10.1038/s41746-022-00617-6 (PMC9198008; doi:10.1038/s41746-022-00617-6)
Supplement: Supplementary file 1 — Supplementary files [file 41746_2022_617_MOESM1_ESM.docx]

Supplementary Table 1

**Supplementary Table 1.** General summarization of reviewed articles.

| **Paper** | **Drug(s) explored** | **EHR data** | **Contribution of EHR** | **Predictive methods** | **Validation methods** | **Results** | **Limitations/future work** |
| --- | --- | --- | --- | --- | --- | --- | --- |
| Goldstein et al ^17^ | Calcium Channel Blockers | 1) 9960 patients with glucose tolerance test (DTT) from Synthetic Derivative (SD), a de-identified image of Vanderbilt University Medical Center’s electronic medical record (EMR), 2) 37,380 patients from BioVU | Prediction (diagnoses, laboratory tests) | Logistic regression, Multivariate regression |  | 6 drug classes were associated with changes in the glucose tolerance test (GTT). L-type calcium channel blocking antihypertensives (CCBs), was associated with a 3.18mg/dL (95% CI −6.18 to −0.18) decrease in glucose during GTT, and Serotonin receptor type 3 (5HT-3) antagonist antinausea medications were associated with a 3.54mg/dL (95% CI 1.86 to 5.23) increase in glucose during GTT. | 1) Data bias, 2) Small sample size, 3) Diagnosis misclassification |
| Xu et al ^18^ | Metformin | 1) 32 415 adults with a cancer diagnosis at Vanderbilt, and 2) 79 258 cancer patients at Mayo from 1995 to 2010 | Validation (drug and mortality) | Cox regression | Validated the potential drugs using stratified Cox proportional hazard models. | Metformin was associated with a 22% decrease in overall mortality compared to other oral hypoglycemic medications (HR 0.78; 95% CI 0.69 to 0.88) and with a 39% decrease compared to type 2 diabetes patients on insulin only (HR 0.61; 95% CI 0.50 to 0.73). Diabetic patients on metformin also had a 23% improved survival compared with non-diabetic patients (HR 0.77; 95% CI 0.71 to 0.85) | 1) Incomplete data, 2) Exposure time misclassification, 3) Data bias |
| Wu et al ^19^ | 146 Drugs from six drug classes (statins, proton pump inhibitors, angiotensin-converting enzyme inhibitors, β-blockers, nonsteroidal anti-inflammatory drugs, and α-1 blockers) | 1) 43,310 adults with a cancer diagnosis at Vanderbilt, and 2) 98,366 cancer patients at Mayo from 1995 to 2010 | Prediction (drug effect on cancer survival) | Cox regression | 1) Search biomedical literature for supporting evidence, 2) Search human interventional cancer trials for supporting evidence | 22 drugs from six drug classes (statins, proton pump inhibitors, angiotensin-converting enzyme inhibitors, β-blockers, nonsteroidal anti-inflammatory drugs, and α-1 blockers) were associated with improved overall cancer survival (false discovery rate, <0.1) from VUMC; nine of the 22 drug associations were replicated at the Mayo Clinic. | 1) Incomplete data, 2) Unmeasured confounding factors |
| Zhou et al ^20^ | Pioglitazone, Liraglutide, Varenicline, Prednisone (amongst others, 30+ presented in the paper) | 72 million patients from the IBM Watson Health Explorys database | Validation (drug and disease) | Network-based | 1) Validated internally based on k-fold cross-validation of drug-target pairs, 2) Validated the potential drugs by clinical trials, 3) Validate the potential drugs by EHR-based case-control studies | The area under the receiver operating characteristic (ROC) curve was 0.97 in the de novo cross-validation when evaluated using 910 drugs. TargetPredict outperformed a state-of-the-art phenome-driven DTI prediction system as measured by precision-recall curves [measured by average precision (MAP): 0.28 versus 0.23, P-value< 0.0001]. The EHR-based case-control studies identified that the prescription's top-ranked repositioned drugs are significantly associated with lower odds of AD diagnosis. | 1) Incomplete data, 2) Data bias, 3) Incomplete knowledge |
| Dang et al ^21^ | Unspecified | 4000 patients extracted from the MIMIC II database | Prediction (drug diagnosis information) | Pairwise mutual information | Evaluated by the literature review | 74.6 % for recall of retrieved new indications | 1) Ineffective for rare drugs |
| Tatonetti et al ^22^ | Unspecified | 1) 1,851,171 adverse event reports in the AERS from the FDA’s Web site from the first quarter of 2004 to the first quarter of 2009, 2) Canada’s MedEffect resource, the sister database to the AERS containing about 300,000 adverse event reports from an independent database of the adverse event reports in AERS of 2009 to 2010 | Prediction (adverse event) | Similarity-based | Validated with DrugBank, Matador, and Psychoactive Drug Screening Program (PDSP) chemical databases | 40 most likely candidate drugs for glaucoma treatment were highlighted based false discovery rate (FDR) | 1) Lack causative inference, 2) Missing data, 3) Data quality, 4) Ineffective for rare events |
| Kim et al ^26^ | 5 Distinct Drug Candidates that increase/decrease hemoglobin A1c (HbA1c), low-density lipoprotein (LDL) cholesterol, triglycerides (TG), and high-density lipoprotein (HDL) cholesterol | Patients in Asan Medical Center, Seoul, Korea, from 2013 to 2017 | Prediction (laboratory tests, drugs) | T-test | Validated with anatomical therapeutic classification (ATC), and literature review. | Among 1774 drugs, 45 were associated with increases in HDL cholesterol, and 41, 146, and 65 were associated with reductions in HbA1c, LDL cholesterol, and TG, respectively. The algorithm had high values for both sensitivity (range, 0.95–1.00) and negative predictive value (range, 0.95–1.00) | 1) Incomprehensive validation, 2) Data bias |
| Paik et al ^27^ | Terbutaline sulfate | 530,000 patients from the tertiary hospital | Prediction (similarity measures for drug- and disease pairs), validation (drug and disease) | 1) Network, and 2) similarity-based | 1) Evaluated based on tenfold cross-validation, 2) Validated the potential therapeutic effect of terbutaline sulfate in an in a vivo zebrafish model of amyotrophic lateral sclerosis | ClinDR outperformed other methods in terms of AUC ROC based on cross-validation. Terbutaline sulfate is discovered as a promising candidate for amyotrophic lateral sclerosis. | 1) Single EHR database |
| Challa et al ^28^ | 14 Distinct Drugs across 7 disease areas | Patients from Vanderbilt University Medical Center | Prediction (billing codes) | 1) Chi-squared test, and 2) Fisher’s exact test |  | 227 ‘‘target-action pairs’’ of the pool of 237 ‘‘pairs’’ demonstrated cross-listed SNPs/SNVs, giving 96% total SNP/SNV coverage for the ExomeChip population in BioVU, is determined. | 1) Small sample size, 2) Incomplete knowledge, 3) Without considering drug toxicity |
| Nordon et al ^29^ | Statins, proton pump inhibitors, and alpha-blockers | 30,705 hypertension patients and 15,893 diabetic patients provided by Maccabi healthcare | Prediction (disease, medication, laboratory tests) | 1) Chi-squared test, and 2) network-based | 1) Validate the result via PubMed publications of small trials, 2) Validated the drug-disease correlation through expert rating | The number of treatment group candidates produced by the algorithm is smaller in magnitude than the baselines approach and provides a more manageable set of candidates for further investigation. Two candidates for the hypertension test case: “statins” and “proton-pump inhibitors” were identified. | 1) Data bias |
| Pinoges et al ^30^ | Antiretroviral therapies (e.g., nucleoside reverse transcriptase inhibitors (NRTI) and/or non-NRTI (NNRTI) drugs) | 2302 patients participated 5 cross-sectional virological and genotype testing studies were conducted in Médecins Sans Frontières-supported HIV programs in Chiradzulu, Malawi; Phnom Penh, Cambodia; Arua, Uganda; and Busia, Kenya | Prediction (drug and mortality), validation (drug and mortality) | Adjusted logistic and Cox-regression models | Validated by multiple sites comparison. | The most frequent nucleoside reverse transcriptase inhibitors (NRTI)-associated mutations were M184V (n = 133), T215Y (n = 23), and M41L (n = 19). 295 non-NRTI (NNRTI)-associated mutations were detected, the most frequent being K103N (n = 63), Y181C (n = 56), and V179I (n = 54). | 1) Missing data， 2) Outcome misclassification, 3) Unmeasured confounding factors |
| Wang et al ^31^ | 114 Distinct Drugs | 136,128 patients from Sichuan Provincial People’s Hospital from 2015 to 2018 | Validation (drug-disease association) | Chi-squared test | Search EHR for candidate drugs and glaucoma | 40 drugs/chemicals most likely to prevent or treat glaucoma were identified by P-value and false discovery rate (FDR) ranking | 1) Incomplete knowledge, 2) Incomprehensive validation |
| Ghalwash et al ^32^ | Unspecified drugs to lower low-density lipoproteins and glycated hemoglobin | 300,000 patients with their medical information from 2002 to 2005. | Prediction (drug effect on lab test results) | Optimization-based | 1) MEDication Indication resource (MEDI) for known drugs, 2) A manual internet search for potential drugs | DELT outperforms the Baseline Regularization (BR) in terms of precision. Top 20 drugs were identified to reduce the level of Glycated hemoglobin (HbA1c) and Low-Density Lipoproteins (LDL) for diabetes-related complications and cardiovascular problems. | 1) Unmeasured confounding factors |
| Liu et al ^33^ | 55 Distinct Drugs | 107.5 million distinct patients in the MarketScan Commercial Claims and Encounters (CCAE) from 2012 to 2017 | Prediction (disease, medication, comorbidity) | Recurrent neural network (LSTM) | 1) Validate the model through treatment effect estimation, 2) Validate the model performance by measuring feature balance | The proposed method performed better than the other three pre-clinical methods. 55 qualified drugs were identified, in which nine drugs yield a beneficial effect on disease outcomes among the sixteen selected significant drug candidates | 1) Incomprehensive validation |
| Cai et al ^34^ | IL6R blockading Drugs | 332,799 participants from Million Veteran Program | Prediction (laboratory tests, diagnosis), validation (cancer patients with PheWAS codes) | 1) Logistic regression, and 2) Linear regression | Validated through medical record review | The IL6R SNP was most strongly associated with a reduced risk of aortic aneurysm phenotypes (odds ratio, 0.87-0.90; 95% CI, 0.84-0.93). | 1) Small sample size, 2) Diagnosis misclassification, 3) Lack of independent validation, 4) Discrepancy of different data sources |
| Xu et al ^35^ | Psychiatric drugs, Antihypertensive Calcium Channel Blockers, 7 drugs to target the AKT-FOXO and AMPK pathways | 7,216 cancer patients and 653 normal collected from RecData Technology Co, Ltd | Validation (drug and lab test) | 1) T-test, 2) Binomial test, 3) Hypergeometric test, 4) DeLong test, and 5) Log-rank test | 1) Calcium blockers predicted to target AKT and AMPK are validated based on EHR, 2) Seven drugs were repositioned to selectively target AKT-FOXO and AMPK and validated by Worm lifespan assay | The csD2G markedly outperformed both ping-pong algorithm (PPA) and multivariate analysis of variance (MANOVA) in drug-target prediction (area under the curve [AUC]: 0.743 for csD2G and 0.676 and 0.659 for PPA; AUC: 0.685 for csD2G, 0.486 for MANOVA; DeLong test p = 0.0367). 1) Category of psychiatric drugs to inhibit the TGF-b pathway, 2) antihypertensive calcium channel blockers to activate AMPK and inhibit AKT pathways, and 7 drugs targeted the AKT-FOXO and AMPK pathways were identified. | 1) Not completely addressing the drug side effects |
| Reznikov et al ^46^ | Antihistamine (i.e., diphenhydramine, hydroxyzine, and azelastine) | 19,000 patients tested for SARS-CoV-2 within the University of California Health System. | Validation (medication, severity, mortality) | Logistic regression | 1) Validated the potential drugs using logistic regression for association analysis, 2) Validated the candidate drugs using laboratory experiments (lentivirus pseudotyped, recombinant spike protein, and the DMSO vehicle) | Diphenhydramine, hydroxyzine, and azelastine were associated with reduced incidence of SARS-CoV-2 positivity. | 1) Unmeasured confounding factors |
| Hsieh et al ^36^ | 22 Distinct Drugs (e.g., azithromycin, atorvastatin, aspirin, acetaminophen, and albuterol) | 34,043 hospitalized COVID-19 patients in Optum deidentified EHR database | Prediction (medication, laboratory tests) | Network-based | 1) Validated the confidence of our knowledge graph embedding via link prediction, 2) Validated the potential drugs using gene expression signature, 3) Validated the potential drugs through in vitro drug screening studies, 4) Validated the potential drugs through population-based study | a Recall (0.21 to 0.44) and a precision (0.04 to 0.18) implying moderate accuracy of the method. Ten (out of 138) drugs were effective (averaged treatment effect among treated or ATT > 0 and p-value < 0.05) in the EHRs. Among the ten positive drugs, our method identified six positive drugs: Acetaminophen (ATT = 0.25), Azithromycin (ATT = 0.18), Atorvastatin (ATT = 0.17), Albuterol (ATT = 0.14), Aspirin (ATT = 0.14), and Hydroxychloroquine (ATT = 0.08). | 1) Treatment misclassification, 2) Unmeasured confounding factors, 3) Discrepancy of different data sources |
| Koren et al ^37^ | Drugs that control blood glucose (e.g., α1‐adrenoceptor antagonists) | 29,540 patients were diagnosed with type 2 diabetes between 2005 and mid‐2016 from electronic medical charts of Maccabi Health Services | Prediction (laboratory tests, drugs) | 1) Chi-squared test, and 2) Logistic regression |  | Alpha 1‐adrenoceptor antagonists were the only drug class that yielded a significantly better success rate in glucose control (61% success rate for the treated group and 53% success for the untreated group. P < 0.0004 and test statistic of 16.7) | 1) Incomprehensive validation |
| Kuang et al ^38^ | 30 Distinct Drugs that control Fasting Blood Glucose (FBG) level | 75,146 patients with 333,907 Fasting blood sugar (FBG) measurements from Marshfield Clinic. | Prediction (laboratory tests, drugs) | Optimization-based | Evaluated based on literature review | 180 drugs generated by the baseline regularization using λ1 = 86 and λ2 = 2.841977 × 10−4 | 1) Incomprehensive validation |
| Kuang et al ^39^ | 20 Distinct Drugs that control Fasting Blood Glucose (FBG) level | 64,515 patients with 219,306 Fasting blood sugar (FBG) measurements from Marshfield Clinic. | Prediction (laboratory tests, drugs) | Optimization-based | Evaluated based on literature review | Baseline Regularization (BR) and ABR outperformed continuous Self-controlled Case Series (CSCCS) and CSCCSA in terms of AUC ROC. One hundred drugs were identified, in which the top 20 were validated. | 1) Incomprehensive validation |
| Kuang et al ^40^ | 40 Distinct Drugs that control Fasting Blood Glucose (FBG) level | 64,515 patients with 219,306 Fasting blood sugar (FBG) measurements from Marshfield Clinic. | Prediction (drug, diagnosis, laboratory tests) | Optimization-based | Evaluated based on literature review | The continuous Self-controlled Case Series (CSCCS) and CSCCSA models achieved promising results in AUCROC and precision at top K. Two hundred drugs were identified, in which the top 40 were validated. | 1) Limited data elements |
| Jang et al ^41^ | Glutathione and edetic-acid highlighted, amongst others discovered | 59,205 patients from National Health and Nutrition Examination Survey (NHANES). | Prediction (disease, medication, labtest) | 1) Wilcoxon rank-sum test, Fisher’s exact test | Validated with Comparative Toxicogenomics Database (CTD), Clinical Trials, and published literature | A total of 121 drugs were identified for type 2 diabetes, 85 for myocardial ischemia, 102 for stroke, 118 for congestive heart failure, and 36 for asthma. A top 15 drugs were further validated with a literature search. | 1) Small sample size, 2) Bias created by an upper stream application, 3) Discrepancy of different data sources, 4) Incomplete knowledge |
| Wen et al ^42^ | 153 Distinct Drugs | 91,934 patients from National Health and Nutrition Examination Survey (NHANES). | Prediction (medication) | 1) Wilcoxon rank-sum test, and 2) Machine-learning/similarity | 1) Validate the method using the repurposing possibility score, precision at K, and Fold-enrichment test. | 392 drugs for 6 important chronic diseases (including asthma, coronary heart disease, congestive heart failure, heart attack, type 2 diabetes, and stroke) were evaluated by precision. | 1) Limited prediction, 2) Discrepancy of different data sources |
| Stenner et al ^43^ | HMG-CoA reductase inhibitors, serotonin receptor agonists, intranasal steroid sprays, and proton-pump inhibitor | 45,000 members from Vanderbilt University Medical Center | Prediction (disease, medication, prescribing behavior, genetic mutation) | 1) Compliance Ratio, and 2) Chi-squared test |  | The significant improvements of proton-pump inhibitors (PPI) were analyzed. | 1) Lack of generalizability, 2) Data quality, 3) Incomprehensive validation |
| Malki et al ^44^ | 13 distinct drugs with 19 drug-gene associations among the 50 most commonly used drugs | 1) 18,306 participants from GoDARTS study, 2) 20,000 from GS:SFHS study, 3) 5,000 from GoSHARE, and 4) 230,000 from UKBB study. | Validation (diagnosis, medication, radiation, surgery, mortality) | Logistic regression | 1) Validated the association using logistic regression for association analysis, 2) Validated the association from existing literature | For 50 commonly used drugs and 162 independent genetic variants, 815 drug-gene interactions (DGIs) with a significance level of P ≤ 0.05 were identified; 8 of these were significant after Bonferroni correction and a further 11 were validated with a literature search. | 1) Lack of data, 2) Discrepancy of different data sources, 3) Without considering drug-drug interaction (concomitant medication) |
| Bi et al ^45^ | Fluoxetine | 238 patients with brain cancer glioblastoma (GBM)from IBM Health MarketScan database (2003-2017) | Prediction (medication, labtest) | T-test | Validated the potential drugs using survival analysis | A significantly increased survival in brain cancer glioblastoma (GBM) patients treated with fluoxetine. The age, sex, and baseline comorbidity score-adjusted hazard ratio of all-cause death in the fluoxetine-treated group were 0.42 (95% confidence interval [CI], 0.20–0.88; p = 0.022). | 1) Data bias |
| Bejan et al ^47^ | 17 distinct drugs | 9,748 patients with COVID-19 at Vanderbilt University Medical Center (VUMC). | Prediction (medication, severity e..g, (1) never hospitalized, (2) hospitalized with mild conditions and without intensive treatment (hospitalized-mild), (3) admitted to the intensive care unit (ICU), (4) on mechanical ventilation, and (5) dead, and comorbidity) | 1) Logistic regression, and 2) Multivariate regression |  | 17 drugs were significantly associated with decreased COVID-19 severity. Previous exposure to two types of 13-valent pneumococcal conjugate vaccines, PCV13 (odds ratio (OR), 0.31, 95% confidence interval (CI), 0.12–0.81 and OR, 0.33, 95% CI, 0.15–0.73), diphtheria toxoid and tetanus toxoid vaccine (OR, 0.38, 95% CI, 0.15 0.93) were significantly associated with a decreased risk of death (primary outcome). Secondary analyses identified several other significant associations showing lower risk for COVID-19 outcomes: acellular pertussis vaccine, 23-valent pneumococcal polysaccharide vaccine (PPSV23), flaxseed extract, ethinyl estradiol, estradiol, turmeric extract, ubidecarenone, azelastine, pseudoephedrine, dextromethorphan, omega-3 fatty acids, fluticasone, and ibuprofen. | 1) Unmeasured confounding factors, 2) Treatment and outcome misclassification, 3) Lack of generalizability |
| Muraki et al ^48^ | Febuxostat | 358 patients from Tottori University Hospital and Fukuoka Tokushukai Medical Center | Prediction (medication, laboratory tests) | 1) Covariate analysis, and 2) Wilcoxon signed-rank test |  | The study provides a precise description of the time course of the UA-lowering effects of febuxostat and quantitatively detects an interhospital difference in the UA baseline level. A covariate analysis indicated that loops and thiazides increased describe uric acid (UA) baseline levels by 7%–14% and 6%–11%, respectively. “hospital” was identified as a significant covariate for the UA baseline level; the baseline level was 7% higher in the city hospital. | 1) Robustness and reliability |
| Cummings et al ^49^ | Dextromethorphan | 18,677 patients from the US Department of Veterans Affairs. | Validation (diagnosis, severity, medication, mortality ) | Linear regression | Validated the potential drugs using Poisson generalized linear model | A relative risk reduction of 34% for all-cause hospitalizations (Relative Risk (RR) 0.66, 95% Confidence Interval (CI) 0.525-0.832) and 40% for respiratory hospitalizations (RR 0.597, 95% CI 0.423-0.843) in patients with influenza treated with dextromethorphan. | 1) Lack of data, 2) Treatment misclassification,3) Lack of generalizability |
| Zhou et al ^50^ | Tramadol, olanzapine, mirtazapine, bupropion, and atomoxetine | 72.9 million patients of IBM Watson Health from 1999 up to August 2020 | Validation (disease, medication) | Network-based | 1) Validate the method by drug ranking and comparing with literature, 2) Validated the potential drugs using the Cochran Mantel Haenszel method | The proposed method prioritized these four drugs (methadone, buprenorphine, naltrexone, and naloxone) within the top 3.4% among 1430 FDA-approved drugs on the network. 17 out of the top 20 are implicated as SUD treatments through different sources. Tramadol, olanzapine, mirtazapine, bupropion, and atomoxetine were associated with increased odds of opioid use disorders (OUD) remission (adjusted odds ratio: 1.51 [1.38–1.66], 1.90 [1.66–2.18], 1.38 [1.31 1.46], 1.37 [1.29–1.46], 1.48 [1.25–1.76], p-value < 0.001, respectively). Genetic and functional analyses showed these five candidate drugs directly target multiple OUD-associated genes including BDNF, CYP2D6, OPRD1, OPRK1, OPRM1, HTR1B, and POMC, SLC6A4, and OUD-associated pathways, including opioid signaling, G protein activation, serotonin receptors, and GPCR signaling. | 1) Data bias, 2) Discrepancy of different data sources, 3) Lack of generalizability, 4) Incomplete knowledge |
| Ozery-Flato et al ^51^ | Rasagiline, zolpidem, azithromycin, and valsartan | 1) 60 million patients from IBM Explorys Therapeutic Dataset, and 2) 120 million patients from IBM MarketScan Research Databases | Prediction (disease, medication) | Causative inference methods | 1) Applied a meta-analysis of estimated effects and assessed the level of agreement between the two different causal inference methods | The effect of 259 drugs on psychosis and dementia was measured based on 1453 emulated trials. Only 4 (0.3%) of the 1,453 trials ended with significant beneficial effects at a false discovery rate (FDR) of 5% by the two causal estimation methods and in both databases. These 4 trials involved 4 distinct drugs: rasagiline, zolpidem, azithromycin, and valsartan. A strong and significant correlation was observed between the effects estimated by the two causal inference methods (focusing on drugs where at least one of the estimated effects is significant at FDR of 5%) | 1) Bias generated in the data processing |
| Bai et al ^52^ | Atorvastatin | 1) 1.8 million adult and pediatric patients seen at Stanford University Medical Center from Jan 1, 2008, to Dec 31, 2015, and 2) 63 million US residents from Jan 1, 2004, to Dec 31, 2016, from Optum Clinformatics DataMart | Prediction (disease, medication, surgery) | Meta-analysis | Validated the potential drugs using Cox proportional hazard model | Atorvastatin treatment had the highest inverse correlation with the UC gene signature among nononcolytic FDA-approved therapies. Atorvastatin intake was significantly associated with a decreased risk of colectomy, a marker of treatment-refractory disease, compared to patients prescribed a comparator drug (Stanford Research Repository (STARR): HR = 0.47, P = 0.03; Optum Clinformatics DataMart: HR = 0.66, P = 0.03), irrespective of age and length of atorvastatin treatment. | 1) Data bias, 2) Lack of data |

Supplementary Method 1

We abstracted the articles with the following flow, 1) journal and articles, 2) data used, 3) methods, and 4) results of repurposing (see Supplementary Figure 1). For journals and articles, we investigated the journal types, the article publication year, and geographic locations of institution of the authors. For data used, we examined the data source (e.g., EHR only, literature, or knowledgebase) and the type of EHR data included (e.g., diagnosis, medication). We investigated the methods designed in three primary ways, 1) data processing, 2) the computational methods to find the drugs for certain diseases or specific targets, and 3) the experiment and validation. For the results of repurposing, we investigated the 1) diseases targeted, 2) drug explored, and 3) data and tools published. With respect to those studies that conducted cross-validation in their experiments, it is less informative to illustrate the performance (e.g., AUC ROC) as the experiment is conducted to alleviate concerns of validation cross-contamination and/or bias in the validation process and, thus, such metrics are not comparable. Therefore, the results of repurposing were focused on the drugs explored in these studies in this survey.

Supplementary Figure 1

| 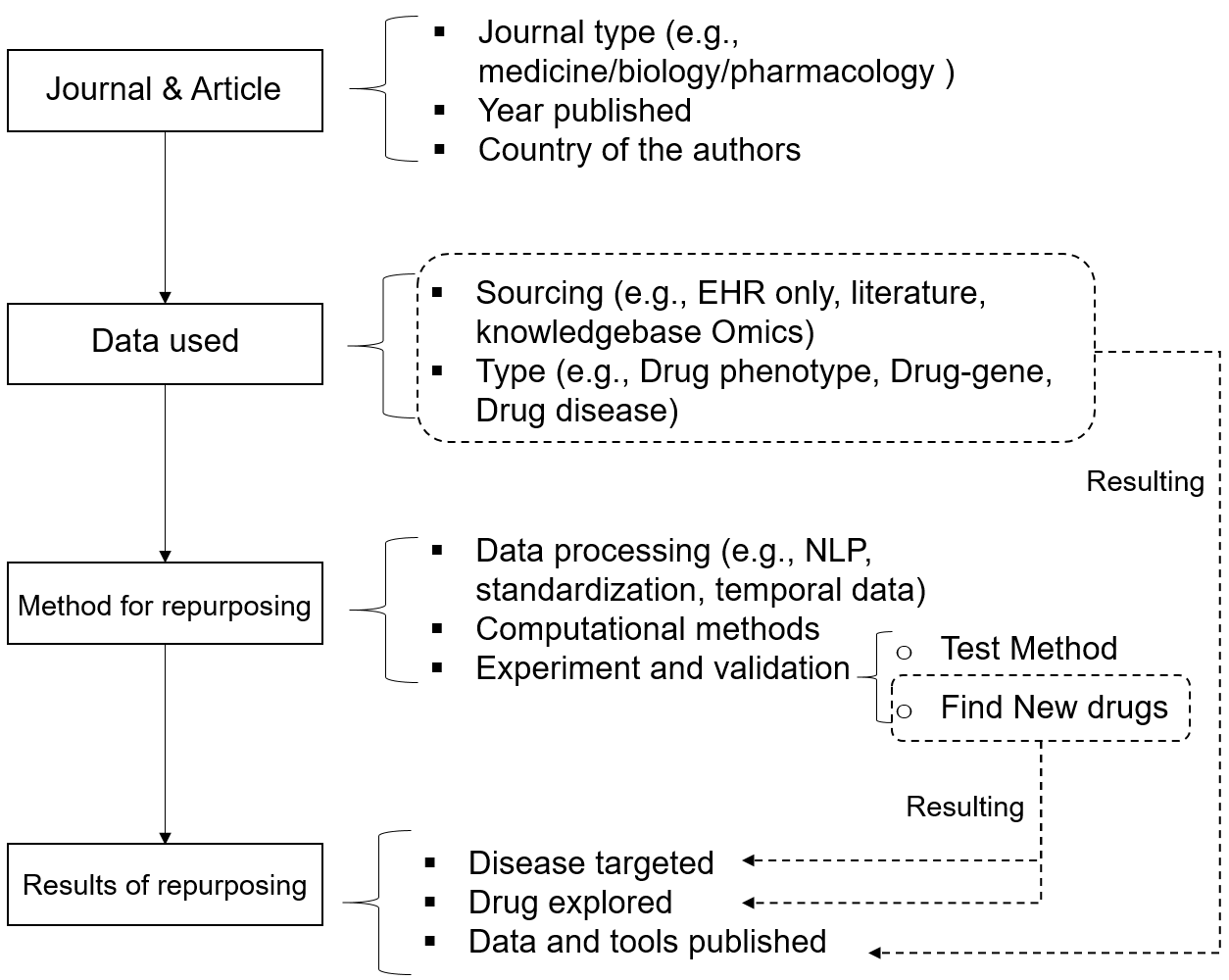 |
| --- |
| **Supplementary Figure 1.** Flow chart for article review. |

Supplementary Figure 2

|  |  |
| --- | --- |
| 1. sources | 1. knowledgebase used |
| **Supplementary Figure 2.** Distribution of sources and knowledgebase. | |

Supplementary Table 2

**Supplementary Table 2.** Summary of articles included that used external data sources.

| **Paper** | **External sources** |
| --- | --- |
| Zhou et al ^20^ | TreatKB, SIDER, UMLS, Disease Comorbidity Network (DCN), STRING database, DrugBank, OMIM |
| Goldstein et al ^17^ | SNP genotyping data from BioVU, DrugBank |
| Wu et al ^19^ | Biomedical literature, Cancer trials indexed in ClinicalTrials.gov |
| Ghalwash et al ^32^ | Drugbank, PubChem, RedBook |
| Xu et al ^35^ | Genomics data, Animal Transcription Factor Database, DGIdb, STITCH, BindingDB, KEGG, Reactome, CMap, DrugBank, ChEBI, USP drug classification system |
| Paik et al ^27^ | DrugBank, CTD, STITCH, HPRD, BioGrid, IntAct, MINT, DIP, GO |
| Wang et al ^31^ | OMIM, GWAS catalog, Phenolyzer database, GEO database, DAVID database, DBIdb, CLUE database, KEGG drug |
| Tatonetti et al ^22^ | SIDER, DrugBank, Matador, Psychoactive Drug Screening Program (PDSP) |
| Cai et al ^34^ | UKBiobank, Vanderbilt University Biobank (BioVU) |
| Pinoges et al ^30^ | Genotype testing |
| Dang et al ^21^ | Drugs.com, DrugBank, Biomedical literature |
| Koren et al ^37^ | Biomedical literature |
| Challa et al ^28^ | DrugBank |
| Khatri et al ^52^ | Omics data |
| Mischel et al ^45^ | Omics data |
| Radinsky et al ^29^ | Biomedical literature |

Supplementary Table 3

**Supplementary Table 3.** Summary of data processing in the studies.

| **Paper** | | | **NLP used** | | **Standardization used** | | **Temporal information used** |
| --- | --- | --- | --- | --- | --- | --- | --- |
| Wu et al ^19^ | | | Medication information was extracted from unstructured (ie, clinical notes) data using MedEx | | RxNorm was used for medication | |  |
| Pinoges et al ^30^ | | |  | |  | | Virological status of patients and all-cause mortality in the 4 years |
| Paik et al ^27^ | | |  | | Proteomics Standard Initiative − Molecular Interactions (PSI-MI) codes; 2) Gene Ontology (GO) from NCBI; 3) ATC codes to drugs, and ICD-10 and OMIM identifiers to diseases | |  |
| Zhou et al ^20^ | | |  | | Disease names were normalized with SNOMED-CT; Individual drug names were normalized with RxNorm; Laboratory tests were normalized by LOINC. | |  |
| Xu et al ^18^ | | | 1) MedEx was used to extract medication names and signature information from unstructured clinical text.  2) a recently developed smoking status extraction NLP algorithm was utilized.  3) a regular expression-based program was developed to extract height and weight information from clinical notes. | |  | |  |
| Dang et al ^21^ | | | The terms indicating drugs and diseases were extracted from the triads of sentences by MetaMap. | |  | |  |
| Challa et al ^28^ | | |  | | Clinical phenotypes were standardized by billing codes. | |  |
| Koren et al ^37^ | | | Weight, age, BMI, and smoking status were extracted from the electronic medical charts. | |  | |  |
| 338  Kim et al ^26^ | | |  | | EHR data were converted to a common data model (CDM) version 5.0.  For a specific laboratory test, every result of each person was labeled as either exposed or unexposed for each drug, and the averages of the labeled values were paired according to the individual drug. | | Only the records with intervals (< 1 year) between the date of drug prescription and laboratory tests were selected for analysis |
| Bejan et al ^47^ | MedXN was used to extract drug information | |  | |  | | |
| Liu et al ^33^ |  | | The drug code (NDC) in MarketScan database were mapped to OMOP standard concept IDs. | | Drug, outcomes temporal sequence data and existing confounding variables were used as the input data for the predictive model | | |
| Nordon et al ^29^ |  | | UMLS was used to standardize the node in the Knowledge graph. | |  | | |
| Ozery-Flato et al ^51^ |  | |  | | Drug temporal data was used to build the cohort for the emulated RCTs | | |

Supplementary Table 4

**Supplementary Table 4.** Summary of computational methods for repurposing drugs.

| **Method Type** | **Method** | **Paper** | **Summary of method** |
| --- | --- | --- | --- |
| Machine learning | Deep learning | Liu et al ^33^ | A high-throughput screening framework was developed for on-market drugs by emulating a randomized clinical trial to evaluate the effectiveness of each drug with existing large-scale real-world data. The framework extracts a list of potential repurposing drug ingredients, identifies the corresponding user and non-user sub-cohorts, computes features and disease progression outcomes for all patients in both sub-cohorts, and estimates the treatment effects using deep learning methods. |
|  | Network | Zhou et al ^20^ | The standard random walker with restart network ranking algorithm was applied to predict potential drug-disease interactions and repositioned drug candidates. |
|  |  | Paik et al ^27^ | Given a drug-disease pair for query, the algorithm found similar drugs and diseases via the similarity matrices, checked the association of similar drug-disease pairs via a bipartite network, and predicted a score for each edge for the drug-disease pair to determine the repositioning ability of the candidate. |
|  |  | Hsieh et al ^36^ | A COVID-19 knowledge graph was developed to represent interactions between SARS-CoV-2 baits, host genes, pathways, targets, drugs, and phenotype using a deep graph neural network (GCN) embedding. GCN was then used to predict the candidate drugs based on the interaction network. |
|  |  | Nordon et al ^29^ | A drug repurposing system was developed by jointly harnessing large-scale electronic health records (EHR) data and a concept graph from the medical literature. An EHR database was mined to discover the correlation between drugs and diseases via a chi-square test of identified case and control groups. The reasoning for the correlation is further induced by a biomedical knowledge graph extracted from a medical literature data repository. |
|  |  | Zhou et al ^50^ | A drug prediction system was developed to model the interconnections among drugs, side effects, and genes. The candidate drugs were predicted using the network-based ranking algorithms. |
|  | Optimization | Ghalwash et al ^32^ | The interactions of drugs and laboratory test results were formulated as a regularized least-square unconstrained convex optimization problem. The least-square objective function modeled the effects of drugs (independent variables) on the levels of laboratory test results (dependent variables) as coefficients of independent variables. |
|  |  | Kuang et al ^40^ | A Continuous Self-controlled Case Series (CSCCS) method was to model the interaction between physiological measurements and drug prescriptions by using a linear fixed-effect model, which was equivalent to solving a least-squares optimization problem. |
|  |  | Kuang et al ^38^ | A baseline regularization (BR) method was developed to model how the potential influence of various drugs over time on the value of fasting blood glucose (FBG) measurements using the baseline regularization, a regularized least square function, to identify potential repurposing opportunities. |
|  |  | Kuang et al ^39^ | A baseline regularization (BR) model was developed to extend the one-way fixed-effect model (a least-square method) by considering the baseline value and its joint effect with drugs and introducing regularization to the baseline model parameters. |
|  | Similarity | Paik et al ^27^ | Given a drug-disease pair for query, the algorithm found similar drugs and diseases via the similarity matrices, checked the association of similar drug-disease pairs via a bipartite network, and predicted a score for each edge for the drug-disease pair to determine the repositioning ability of the candidate. |
|  |  | Tatonetti et al ^22^ | The pairwise similarity metrics between all drugs in the side effect databases were computed to predict new drug indications. The side-effect similarity between the two drugs was measured by computing the Tanimoto coefficient between their side-effect profiles and its z-score normalization. |
|  |  | Wen et al ^42^ | Clinical drug effect vectors were built with a continuous self-controlled case series model on longitudinal electronic health record data. The clinical disease sign vectors were created using a Wilcoxon rank-sum test on large-scale national survey data. The repurposing possibility score for each drug-disease pair was calculated via a dot product-based scoring function on clinical disease sign vectors and clinical drug effect vectors. |
| Statistical analysis | Adjusted logistic | Pinoges et al ^30^ | In cross-sectional analyses, multivariable logistic regression was used to investigate risk factors for resistance. In longitudinal analyses, multivariable proportional Cox models were fitted to investigate the association between the virological status of patients and all-cause mortality in the following 4 years and to assess associations with other individual-level factors. |
|  | Binomial test | Xu et al ^35^ | The drug-gene interaction (DGIs) were generated by drug-to-gene normalized enrichment score (d2gNES) based on up-and downregulated core signatures (CSS) of human genes. |
|  | Causal inference methods | Ozery-Flato et al ^51^ | A framework for Randomized Controlled Trial (RCT) emulation was developed identifying drug repurposing candidates from observational healthcare data. The RCT emulator identifies a list of repurposing candidates, assigns patients to the respective cohorts for each candidate, computes a baseline and follow-up attributes for patients in both cohorts, and estimates its effect on disease outcomes in the treatment versus control cohorts via causal inference. |
|  | Chi-squared test | Koren et al ^37^ | Logistic regression was used for predicting the probability of treatment success of a specific drug (the propensity score). Pearson's chi-squared test was used to determine whether a specific drug treatment achieved independently higher success rates. |
|  |  | Wang et al ^31^ | The p-values of the chi-squared test for drugs targeted to glaucoma genes and diseases were calculated. |
|  |  | Challa et al ^28^ | The algorithm integrated the drug data and single-nucleotide polymorphism(SNP)/single nucleotide variant (SNV) data through target genomics for generating a shortlist of candidates for PheWAS call quality for prioritizing repurposable drug candidates. |
|  |  | Nordon et al ^29^ | A drug repurposing system was developed by jointly harnessing large-scale electronic health records (EHR) data and a concept graph from the medical literature. An EHR database was mined to discover the correlation between drugs and diseases via a chi-square test of identified case and control groups. The reasoning for the correlation is further induced by a biomedical knowledge graph extracted from a medical literature data repository. |
|  |  | Stenner et al ^42^ | The Compliance Ratio (CR) was calculated by dividing the number of prescriptions with recommended therapeutic interchange medications by the number of prescriptions with non-recommended medications to measure effectiveness. The Chi-squared tests were used to explore statistical significance. |
|  | Compliance ratio | Stenner et al ^43^ | The Compliance Ratio (CR) was calculated by dividing the number of prescriptions with recommended therapeutic interchange medications by the number of prescriptions with non-recommended medications to measure effectiveness. The Chi-squared tests were used to explore statistical significance. |
|  | Covariate analysis | Muraki et al ^48^ | For approach A, structural models and covariates between the two hospitals were compared. For approach B, differences between the 2 hospitals were evaluated by performing a covariate analysis on all population pharmacodynamic (PPD) parameters. In addition, differences between the 2 hospitals were evaluated using the Wilcoxon signed-rank test. |
|  | Cox regression | Xu et al ^18^ | Cox regression models were used to determine the influence of metformin on cancer mortality. |
|  |  | Wu et al ^19^ | Cox proportional hazards regression modeling was applied to measure the association of drug exposure with cancer survival. |
|  |  | Pinoges et al ^30^ | In cross-sectional analyses, multivariable logistic regression was used to investigate risk factors for resistance. In longitudinal analyses, multivariable proportional Cox models were fitted to investigate the association between the virological status of patients and all-cause mortality in the following 4 years and to assess associations with other individual-level factors. |
|  | DeLong test | Xu et al ^35^ | The drug-gene interaction (DGIs) were generated by drug-to-gene normalized enrichment score (d2gNES) based on up-and downregulated core signatures (CSS) of human genes. |
|  | Fisher’s exact test | Challa et al ^28^ | The algorithm integrated the drug data and single-nucleotide polymorphism(SNP)/single nucleotide variant (SNV) data through target genomics for generating a shortlist of candidates for PheWAS call quality for prioritizing repurposable drug candidates. |
|  |  | Jang et al ^41^ | A score for each disease-drug pair was calculated by a comparison of complementarity and association between the clinical disease signature and drug effect vectors. The clinical disease signature vector was obtained from the Wilcoxon rank-sum test. The clinical drug effect vector was obtained from Fisher’s exact test. |
|  | Hypergeometric test | Xu et al ^35^ | The drug-gene interaction (DGIs) were generated by drug-to-gene normalized enrichment score (d2gNES) based on up-and downregulated core signatures (CSS) of human genes. |
|  | Linear regression | Cai et al ^34^ | The phenome-wide association study (PheWAS) used logistic regression models for testing associations of the IL6R SNP with phenotype groups and linear regression models for testing associations of the IL6R SNP with laboratory measurements. |
|  |  | Cummings et al ^49^ | A Poisson generalized linear model was used to estimate the relative risk for all-cause and respiratory hospitalizations between treated patients and untreated patients to identify potential drugs for influenza. |
|  | Logistic regression | Cai et al ^34^ | The phenome-wide association study (PheWAS) used logistic regression models for testing associations of the IL6R SNP with phenotype groups and linear regression models for testing associations of the IL6R SNP with laboratory measurements. |
|  |  | Koren et al ^37^ | Logistic regression was used for predicting the probability of treatment success of a specific drug (the propensity score). Pearson's chi-squared test was used to determine whether a specific drug treatment achieved independently higher success rates. |
|  |  | Goldstein et al ^17^ | The SNP genotyping data were identified for the patients with gestational diabetes and type 2 diabetes and their association with drug candidates were analyzed using logistic regression. The effect of drug exposure on glucose tolerance test (GTT) values was examined with multivariate regression. |
|  |  | Malki et al ^44^ | A logistic regression model for the drug-stop or dose-decrease phenotype was used together with a log-additive genetic model to identify the associations between the genetic variants and both phenotypes for all drugs. |
|  |  | Reznikov et al ^46^ | The antihistamine candidates for COVID-19 repurposing were identified by mining electronic health records of a large number of patients tested for SARS-CoV-2. Logistic regression was used for association analysis between candidates and COVID-19 by measuring odds ratio, confidence interval, and p-value. |
|  |  | Bejan et al ^47^ | Drug-wide association studies were performed on the patients with COVID-19 in the electronic health records to identify potential treatment for COVID-19. Multivariable logistic regression was applied to estimate the effect of drug exposure on COVID-19 disease outcomes with overlap weighting using propensity score. |
|  | Log-rank test | Xu et al ^35^ | The drug-gene interaction (DGIs) were generated by drug-to-gene normalized enrichment score (d2gNES) based on up-and downregulated core signatures (CSS) of human genes. |
|  | Meta-analysis | Bai et al ^52^ | A multi-cohort meta-analysis was performed for colon biopsy transcriptome samples from publicly available datasets to identify a robust disease gene signature. The gene signature was compared to transcriptomic profiles induced by FDA-approved drugs to identify potential drug targets for Ulcerative colitis. |
|  | Multivariate regression | Goldstein et al ^17^ | The SNP genotyping data were identified for the patients with gestational diabetes and type 2 diabetes and their association with drug candidates were analyzed using logistic regression. The effect of drug exposure on glucose tolerance test (GTT) values was examined with multivariate regression. |
|  |  | Bejan et al ^47^ | Drug-wide association studies were performed on the patients with COVID-19 in the electronic health records to identify potential treatment for COVID-19. Multivariable logistic regression was applied to estimate the effect of drug exposure on COVID-19 disease outcomes with overlap weighting using propensity score. |
|  | Pairwise mutual information | Dang et al ^21^ | The drug-disease pairs were extracted from the clinical notes and determined by using pairwise mutual information and step activation function. |
|  | T-test | Xu et al ^35^ | The drug-gene interaction (DGIs) were generated by drug-to-gene normalized enrichment score (d2gNES) based on up-and downregulated core signatures (CSS) of human genes. |
|  |  | Kim et al ^26^ | A one-sample t-test with the Bonferroni correction was conducted for the estimation of the effect of an individual drug on specific laboratory tests. |
|  |  | Bi et al ^45^ | A statistical t-test was applied to compare the case and control groups identified from the real-world electronic medical record to measure a significant difference of prolongs survival of patients. |
|  | Wilcoxon rank-sum test | Jang et al ^41^ | A score for each disease-drug pair was calculated by a comparison of complementarity and association between the clinical disease signature and drug effect vectors. The clinical disease signature vector was obtained from the Wilcoxon rank-sum test. The clinical drug effect vector was obtained from Fisher’s exact test. |
|  |  | Wen et al ^42^ | Clinical drug effect vectors were built with a continuous self-controlled case series model on longitudinal electronic health record data. The clinical disease sign vectors were created using a Wilcoxon rank-sum test on large-scale national survey data. The repurposing possibility score for each drug-disease pair was calculated via a dot product-based scoring function on clinical disease sign vectors and clinical drug effect vectors. |
|  | Wilcoxon signed-rank test | Muraki et al ^48^ | For approach A, structural models and covariates between the two hospitals were compared. For approach B, differences between the 2 hospitals were evaluated by performing a covariate analysis on all population pharmacodynamic (PPD) parameters. In addition, differences between the 2 hospitals were evaluated using the Wilcoxon signed-rank test. |

Supplementary Table 5

**Supplementary Table 5.** Summary of evaluation for repurposing drugs.

| **Paper** | **Evaluation metrics** | **Object of evaluation** | **Training source (learning information)** | **Validation source** |
| --- | --- | --- | --- | --- |
| Wang et al ^31^ | True positive | Performance of method | 1) GWAS Catalog, OMIM, Phenolyzer, GEO, and published papers (gene information)  2) DAVID (pathway information)  3) PheWAS database (disease-gene information)  4) DGIdb, KEGG, and CLUE (drug/chemical gene associations) | EHR |
| Wu et al ^19^ | True positive | Performance of method | EHR (drug effect on cancer survival) | 1) Literature  2) Clinical trials |
| Pinoges et al ^30^ | True positive | Performance of method | EHR (drug resistance and mortality) | EHR |
| Paik et al ^27^ | 1) AUC ROC  2) Sensitivity  3) Specificity | 1) Performance of method  2) Validation of New drugs | 1) EHR (similarity measures for drug- and disease pairs)  2) Genomic data (similarity measures for drug- and disease pairs) | 1) EHR  2) Laboratory experiment |
| Ghalwash et al ^32^ | Precision | 1) Performance of method  2) Validation of new drugs | 1) Drugbank (drug names)  2) PubChem (chemical substructures)  3) RedBook(therapeutic classes)  4) EHR (drug effect on lab test results) | 1)Knowledge base  2) Internet search |
| Zhou et al ^20^ | 1) AUC ROC  2) Precision  3) Recall  4) P-value | 1) Performance of method  2) Validation of new drugs | 1) Context-sensitive phenotypic drug network (drug–side effect information)  2) Disease comorbidity network (disease information)  3) Drug–disease treatment knowledge base (drug-disease information)  4) Protein-protein interaction network (protein-protein interaction information) | 1) Knowledge base  2) EHR  3) Clinical trials |
| Xu et al ^18^ | P-value | Validation of new drugs | No training reported | EHR |
| Xu et al ^35^ | 1) AUC ROC  2) True positive | 1) Performance of method  2) Validation of new drugs | 1) KEGG pathway (human signal transduction pathway gene sets)  2) Reactome (human signal transduction pathway gene sets)  3) Animal Transcription Factor Database (human transcription factors, enzymes, transporters,  receptors and ion channels)  4) Human DEPhOsphorylation Database (human transcription factors, enzymes, transporters,  receptors and ion channels)  5) IUPHAR/BPS database (human transcription factors, enzymes, transporters,  receptors and ion channels)  6) TCGA (RNA-seq data)  7) CMap (Drug treatment microarray data)  8) DrugBank (Drug gene information)  9) DGIdb (Drug gene information)  10) STITCH (Drug gene information)  11) BindingDB (Drug gene information)  12) NCI-60 (gene expression and drug responses) | 1) Knowledge base  2) EHR  3) Laboratory experiment |
| Cai et al ^34^ | Positive predictive value | Performance of method | 1) EHR (laboratory tests, diagnosis)  2) Biobank data | EHR |
| Dang et al ^21^ | 1) Precision  2) Recall  3) F-score | Performance of method | EHR (drug diagnosis information) | Literature |
| Kuang et al ^39^ | 1) AUC ROC  2) Precision | Performance of method Validation of new drugs | EHR (laboratory tests, drugs) | Literature |
| Kuang et al ^40^ | 1) AUC ROC  2) Precision | Performance of method Validation of new drugs | EHR (laboratory tests, drugs) | Literature |
| Kim et al ^26^ | 1) Sensitivity  2) Specificity  3) Negative predictive value  4) Positive predictive value  5) Accuracy | Performance of method  Validation of new drugs | EHR (laboratory tests, drugs) | 1) Knowledge-based  2) Literature |
| Jang et al ^41^ | 1) P-value  2) True positive | Performance of method | 1) EHR (drug, diagnosis, laboratory tests)  2) Literature (PubMed abstracts and their MeSH information)  3) Drugbank (drug information) | 1) Knowledgebase  2) Clinical trials  3) Literature |
| Tatonetti et al ^22^ | AUC ROC | Performance of method | 1) AERS (adverse event)  2) SIDER (adverse event)  3) Canada’s MedEffect resource (adverse event)  4) EHR (adverse event)  5) DrugBank (drug target information)  6) Matador (drug target information)  7) Psychoactive Drug Screening Program (PDSP) chemical  databases (drug target information) | Knowledgebase |
| Bai et al ^52^ | 1) Hazard ratios 2) P-value | Validation of new drugs | 1) NCBI Gene Expression Omnibus (colon biopsy samples)  2) EHR | 1) Claims |
| Bi et al ^45^ | 1) Hazard ratios 2) P-value | Validation of new drugs |  | 1) EHR 2) Claims |
| Cummings et al ^49^ | 1) Relative risk 2) P-value | Validation of new drugs | EHR (demographics, administrative claims, and pharmacy dispensation) | EHR |
| Hsieh et al ^36^ | 1) AUROC 2) Precision 3) Recall 2) Averaged treatment effect among treated | 1) Performance of method  2) Validation of new drugs | Comparative Toxicogenomics Database (drug-target interactions, pathways, gene/drug-phenotype interactions) PPI experimental study (SARS- CoV-2 and host PPIs) | 1) Connectivity Map (cMAP) database 2) Literature 3) EHR |
| Liu et al ^33^ | 1) Treatment effect estimation 2) Confidence intervals 3) P-value | Performance of method | Claims (demographic charac- teristics, diagnosis codes, and prescription medication) | Claims |
| Malki et al ^44^ | 1) Odds ratio 2) P-value | Validation of new drugs | 1) EHR (candidate common drugs) 2) DrugBank (candidate genetic variants) | 1) DrugBank 2) Literature |
| Nordon et al ^29^ | Precision | 1) Performance of method  2) Validation of new drugs | 1) PubMed (biomedical knowledge graph) | 1) PubMed 2) Domain experts |
| Ozery-Flato et al ^51^ | 1) P-value 2) False discovery rate | Performance of method | EHR | Claims |
| Reznikov et al ^46^ | 1) Odds ratio 2) P-value | Validation of new drugs | EHR (medical history and SARS-CoV-2 infection tests) | Laboratory experiment |
| Wen et al ^42^ | 1) Precision at K 2) Fold enrichment test | Performance of method | 1) National Health and Nutrition Examination Survey (questionnaire and laboratory results) 2) EHR (prescription and laboratory results) | Side Effect Resource |
| Zhou et al ^50^ | 1) Odds ratio 2) P-value | 1) Performance of method  2) Validation of new drugs | 1) Side Effect Resource (SIDER) database (drug side effects network) 2) STRING (protein-protein interaction network) | EHR |
| Kuang et al ^38^ | 1) Fasting blood sugar (FBG) level | Validation of new drugs | EHR | Literature |

Supplementary Table 6

**Supplementary Table 6.** Summary of the targeted disease.

| Paper | Disease targeted |
| --- | --- |
| Pinoges et al ^30^ | HIV |
| Paik et al ^27^ | Generic disease, in vivo validation on amyotrophic lateral sclerosis |
| Muraki et al^48^ | Hyperuricemia |
| Wang et al ^31^ | Glaucoma |
| Wu et al ^19^ | Cancer |
| Zhou et al ^20^ | The developed method could be used for 1059 diseases. In this paper, the authors applied the proposed method for identifying new repositioned candidate drugs for Alzheimer’s disease (AD). |
| Ghalwash et al ^32^ | No targeted diseases but relevant test results: low-density lipoprotein and glycated hemoglobin (HbA1c). Low-Density Lipoproteins are a risk factor for cardiovascular and vascular diseases. Higher levels of HbA1c are associated with a high risk of developing diabetes-related complications. |
| Xu et al ^18^ | Cancer |
| Xu et al ^35^ | Cancer |
| Cai et al ^34^ | Cardiovascular Disease |
| Dang et al ^21^ | General |
| Challa et al ^28^ | General |
| Koren et al ^37^ | Type 2 diabetes |
| Kuang et al ^38^ | Control Fasting Blood Glucose (FBG) level for diabetes |
| Kuang et al ^39^ | Control Fasting Blood Glucose (FBG) level for diabetes |
| Kuang et al ^40^ | Control Fasting Blood Glucose (FBG) level for diabetes |
| Kim et al ^26^ | Diabetes and dyslipidemia |
| Jang et al ^41^ | Asthma, Type 2 diabetes, Congestive heart failure, myocardial ischemia, and Stroke |
| Goldstein et al ^17^ | Gestational Diabetes |
| Stenner et al ^43^ | General |
| Tatonetti et al ^22^ | General |
| Bai et al ^52^ | Ulcerative colitis |
| Nordon et al ^29^ | Hypertension, diabetes |
| Bejan et al ^47^ | COVID-19 |
| Bi et al ^45^ | Glioblastoma |
| Cummings et al ^49^ | Influenza |
| Hsieh et al ^36^ | COVID‑19 |
| Liu et al ^33^ | Coronary artery disease |
| Malki et al ^44^ | Chronic conditions |
| Ozery-Flato et al ^51^ | Parkinson’s disease |
| Reznikov et al ^46^ | COVID‑19 |
| Wen et al ^42^ | Asthma, Coronary heart disease, Congestive heart failure, Heart attack, and stroke, Type 2 diabetes |
| Zhou et al ^50^ | Opioid use disorders |

Supplementary Note 1

**Embase: 243**

1     electronic medical record/ or electronic medical record system/

2     (((electronic or computer*) adj2 record*) and ((health or medical) adj2 record*)).ab,kw,ti.

3     (emr or ehr).ab,kw,ti.

4     1 or 2 or 3

5     drug repositioning/

6     ((drug* or medication* or pharm*) adj2 (repurpos* or re-purpos* or rescu* or reposition* or re-position* or reprofil* or re-profil* or discover* or develop* or mine* or mining)).ab,kw,ti. (140361)

7     5 or 6

8     4 and 7

9     limit 8 to english language

10     limit 9 to yr="2000 -Current"

**Medline: 209**

1     exp Medical Records Systems, Computerized/

2     (((electronic or computer*) adj2 record*) and ((health or medical) adj2 record*)).ab,kw,ti.

3     (emr or ehr).ab,kw,ti.

4     1 or 2 or 3

5     drug repositioning/

6     ((drug* or medication* or pharm*) adj2 (repurpos* or re-purpos* or rescu* or reposition* or re-position* or reprofil* or re-profil* or discover* or develop* or mine* or mining)).ab,kw,ti.

7     5 or 6

8     4 and 7

9     limit 8 to english language

10     limit 9 to yr="2000 -Current"

**Scopus:** **263**

( ( ( TITLE-ABS-KEY ( ( electronic  OR  computer* )  W/2  record* )  OR  TITLE-ABS-KEY ( emr  OR  ehr ) ) )  AND  ( TITLE-ABS-KEY ( ( drug*  OR  medication*  OR  pharm* )  W/2  ( repurpos*  OR  re-purpos*  OR  rescu*  OR  reposition*  OR  re-position*  OR  reprofil*  OR  re-profil*  OR  discover*  OR  develop*  OR  mine*  OR  mining ) ) ) )  AND  ( TITLE-ABS-KEY ( ( health  OR  medical )  W/2  record* ) )  Limit to English, 2000+

**Web of Science:** **27**
TOPIC:  ((electronic or computer*)  NEAR/2  record*)  AND  TOPIC:  ((health or medical)  NEAR/2  record*) AND (TOPIC:((drug* or medication* or pharm*)  NEAR/2  (repurpos* or re-purpos* or rescu* or reposition* or re-position* or reprofil* or re-profil* or discover* or develop* or mine* OR mining) ))  Limit to English, 2000+
